# Local Controlled Release of Polyphenol Conjugated with Gelatin Facilitates Bone Formation

**DOI:** 10.3390/ijms160614143

**Published:** 2015-06-23

**Authors:** Yoshitomo Honda, Tomonari Tanaka, Tomoko Tokuda, Takahiro Kashiwagi, Koji Kaida, Ayato Hieda, Yasuyuki Umezaki, Yoshiya Hashimoto, Koichi Imai, Naoyuki Matsumoto, Shunsuke Baba, Kimishige Shimizutani

**Affiliations:** 1 Institute of Dental Research, Osaka Dental University, 8-1 Kuzuhahanazonocho, Hirakata, Osaka 573-1121, Japan; E-Mail: shimizu@cc.osaka-dent.ac.jp; 2 Graduate School of Science and Technology, Kyoto Institute of Technology, Matsugasaki, Sakyo-ku, Kyoto 606-8585, Japan; 3 Department of Orthodontics, Osaka Dental University, 8-1, Kuzuhahanazonocho, Hirakata, Osaka 573-1121, Japan; E-Mails: tocco.tmk.26@gmail.com (T.To.); naoyuki@cc.osaka-dent.ac.jp (N.M.); 4 Department of Oral Implantology, Osaka Dental University; 8-1 Kuzuhahanazonocho, Hirakata, Osaka, 573-1121, Japan; E-Mails: kasiwagi@cc.osaka-dent.ac.jp (T.K.); kaida-k@cc.osaka-dent.ac.jp (K.K.); hieda-a@cc.osaka-dent.ac.jp (A.H.); umezaki@cc.osaka-dent.ac.jp (Y.U.); baba-s@cc.osaka-dent.ac.jp (S.B.); 5 Department of Biomaterials, Osaka Dental University; 8-1 Kuzuhahanazonocho, Hirakata, Osaka 573-1121, Japan; E-Mails: yoshiya@cc.osaka-dent.ac.jp (Y.H.); imai@cc.osaka-dent.ac.jp (K.I.); 6 Department of Oral Radiology, Osaka Dental University, 8-1 Kuzuhahanazonocho, Hirakata, Osaka 573-1121, Japan

**Keywords:** catechin, EGCG, bone formation, gelatin, mesenchymal stem cells

## Abstract

Catechins are extensively used in health care treatments. Nevertheless, there is scarce information about the feasibility of local administration with polyphenols for bone regeneration therapy, possibly due to lack of effective delivery systems. Here we demonstrated that the epigallocatechin-3-gallate-conjugated gelatin (EGCG/Gel) prepared by an aqueous chemical synthesis using 4-(4,6-dimethoxy-1,3,5-triazin-2-yl)-4-morpholinium chloride (DMT-MM) gradually disintegrated with time and facilitated bone formation in a critical size defect of a mouse calvaria. Conjugation of EGCG with the Gel generated cross-linking between the two molecules, thereby leading to a retardation of the degradation of the EGCG/Gel and to a delayed release of EGCG. The prepared EGCG/Gels represented significant osteogenic capability compared with that of the uncross-linked Gel and the cross-linked Gel with uncombined-EGCG. *In vitro* experiments disclosed that the EGCG/Gel induced osteoblastogenesis of a mouse mesenchymal stem cell line (D1 cells) within 14 days. Using fluorescently-labeled EGCG/Gel, we found that the fraction of EGCG/Gel adsorbed onto the cell membrane of the D1 cells possibly via a Gel-cell interaction. The interaction might confer the long-term effects of EGCG on the cells, resulting in a potent osteogenic capability of the EGCG/Gel *in vivo*. These results should provide insight into local controlled release of polyphenols for bone therapy.

## 1. Introduction

Drug delivery systems (DDS) to convey drugs to specific sites are recognized as a prospective method to solve the limitation of conventional and systematic therapies, such as low drug efficacy, poor bioavailability, biodistribution, drug overdose, and toxicity [1,2]. To circumvent undesirable complications and cost issues, locally controlled DDS have particularly gained attention for use in the medical fields such as dentistry [3,4] and regenerative therapies [3,5]. So far, various drugs, such as growth factors [6], miRNA [7], and small molecules [4], have been widely investigated with this technique.

Secondary metabolites of natural polyphenols in all vascular plants consist of simple molecules to complex structures, which have benzene rings in common [8]. Epigallocatechin-3-gallate (EGCG), the most abundant polyphenol in green tea catechin, has attracted significant attention as a health-promoting agent. This polyphenol is known to modulate multiple signaling pathways, including ERK [9], JNK [10,11], MEK1/2 [10], STAT 3 [10,12], and PI3K/Akt [9,10,13], and to cause cellular responses, including (a) cell proliferation [14]; (b) cell differentiation [13,14,15]; (c) cytokine production [11,12]; and (d) cell survival [9]. Consequently, the effects of EGCG in medical uses have been extensively investigated for various diseases, such as cancer [12], metabolic syndrome [16], oral disease [17], cardiovascular disease [18], and bone disease [19,20]. To increase its efficacy, EGCG has been combined with artificial [21,22] and natural polymers [23,24].

As for the bone field, compared with numerous reports evaluating the osteogenic properties of EGCG for cells *in vitro* [14,15,25,26,27], limited studies have addressed the utilization of the catechin for local bone regeneration. To date, there has been a report by Rodriguez *et al.* stating that EGCG mixed with alpha tricalcium phosphate particles enhances bone formation in a rat non-critical size defect. Additionally, the study suggests that higher doses of EGCG conversely suppress bone formation and that a simple combination of EGCG and calcium phosphate readily induces a burst of reagents [19]. In other studies *in vitro*, it has been shown that the capability of EGCG to differentiate cells into osteoblasts decreases from a certain concentration [14,27]. These results suggest that an overdose of EGCG at a local site may suppress bone formation. A system designed to release EGCG at a preferable level may be essential to utilize EGCG for local bone formation.

Gelatin (Gel), a partially hydrolyzed product of collagen, is often applied as a biopolymer for drug delivery carriers as films [28,29] and sponges [29,30], *etc.*, because of its low level of immunogenicity and cytotoxicity [29]. Cross-linked Gel is known to gradually biodegrade *in vivo* [31], which is a preferable characteristic for drug delivery. Taking into account these backgrounds of EGCG and Gel, we hypothesize that a biodegradable polymer conjugated with EGCG may elicit a local controlled release of the catechin via degradation of the complex, resulting in bone formation *in vivo* at a low catechin dose.

In the present study, we fabricated EGCG-conjugated Gels (EGCG (N)/Gel, *N* = the weight percent of EGCG to Gel) through a sponge preparation by an aqueous chemical synthesis and investigated whether the complex induced bone formation in a critical size defect of a mouse calvaria. To elucidate the detailed mechanisms of its osteogenic capability, we further investigated the effects of the EGCG/Gel for osteoblastogenesis of a mouse mesenchymal stem cells line (D1 cells) *in vitro*. The stimulus process of the EGCG/Gel was partially determined using a fluorescein-isothiocyanate (FITC)-labeled EGCG/Gel.

## 2. Results

### 2.1. Synthesis of EGCG/Gels

**Figure 1:**
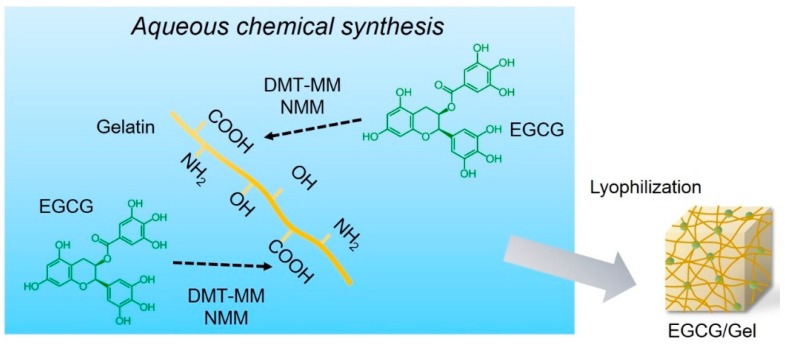
Schematic diagram of the epigallocatechin-3-gallate/gelatin complex (EGCG/Gel) preparation. EGCG/Gels were synthesized using a chemical reaction in water with 4-(4,6-dimethoxy-1,3,5-triazin-2-yl)-4-methyl-morpholinium chloride (DMT-MM) and *N*-methylmorpholine (NMM).

EGCG/Gels were prepared by an aqueous synthesis using various amounts of a water-soluble dehydrative condensing agent, 4-(4,6-dimethoxy-1,3,5-triazin-2-yl)-4-methyl-morpholinium chloride (DMT-MM), and *N*-methylmorpholine (NMM) (Figure 1 and Table 1). The aqueous solution of the Gel, EGCG, DMT-MM, and NMM was stirred for 24 h at room temperature in the dark. The product was purified by dialysis and lyophilized to form a spongy material.

**Table 1:** Synthesis of EGCG/Gels. #: Nubmer.

Sample #	Sample Name	Gelatin (mg)	EGCG (mg)	DMT-MM (mg)	NMM (μL)	Form after Lyophilization	Note
1	Gel	100	0	0	0	Sponge	Readily dissolved in water
2	–	100	0	13.8	5.5	Sponge	Readily dissolved in water
3	–	100	0.13	69.2	27.5	Sponge	Readily dissolved in water
4	–	100	0.13	138	55	Sponge	Readily dissolved in water
5	EGCG(0.7)/Gel	100	0.7	69.2	27.5	Sponge	
6	–	100	6.7	13.8	5.5	Sponge	
7	EGCG(6.7)/Gel	100	6.7	69.2	27.5	Sponge	
8	–	100	67	138	55	Powder	

**Figure 2:**
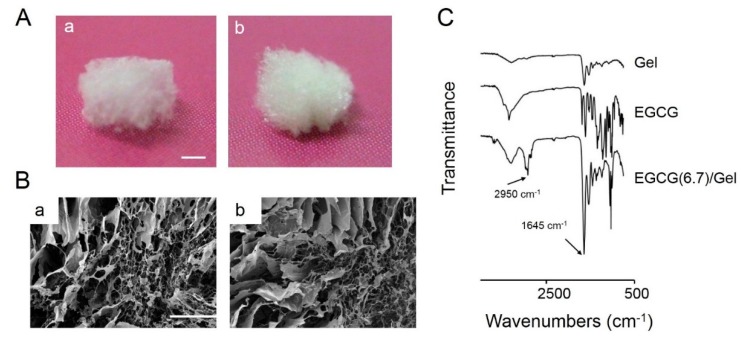
(**A**) Representative macroscopic and (**B**) scanning electron microscopic images of the Gel and EGCG/Gel for (**a**) Gel and (**b**) EGCG (6.7)/Gel; and (**C**) Infrared spectra of Gel, EGCG, and EGCG (6.7)/Gel. Bars: (**A**) 1 mm and (**B**) 300 μm.

Adding excess amounts of EGCG, DMT-MM, and NMM to the Gel yielded powdered EGCG/Gels, whereas an adequate amount of those compounds generated spongy samples (Figure 2A). There was no obvious difference at the macro morphology between the spongy Gel and the EGCG/Gels. Both samples were constructed of numerous macro pores observed by field emission scanning electron microscopy (FE-SEM) (Figure 2B). Figure 2C shows the infrared spectra of the Gel, EGCG, and EGCG (6.7)/Gel. The infrared spectrum of the EGCG (6.7)/Gel had peaks from both Gel and EGCG. The peaks at around 2950 and 1645 cm^−1^ were due to amide and ester linkages from the Gel-Gel and Gel-EGCG, respectively. The results suggest that the prepared EGCG/Gels successfully contained both EGCG and Gel.

### 2.2. Degradation of EGCG/Gels

**Figure 3:**
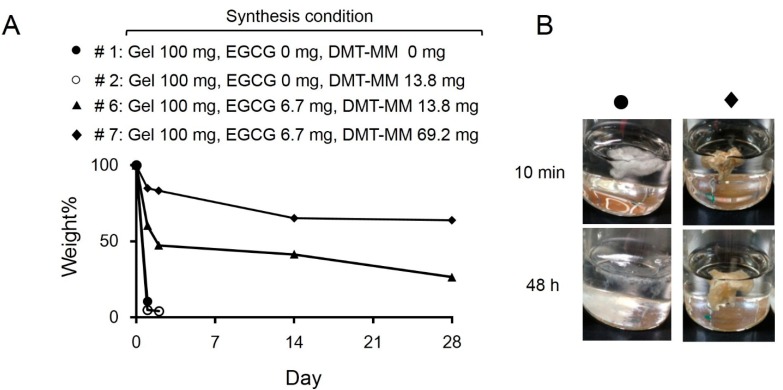
Disintegration of the Gel and EGCG/Gels (See Table 1 for detailed synthesis conditions). (**A**) Weight change of the samples in water after continuous shaking for up to 28 days; (**B**) Representative morphological change of samples *#*1 (Gel) and #7 (EGCG (6.7)/Gel) after 48 h of shaking in water.

To evaluate the robustness of the products, the EGCG/Gels were shaken in water for up to 28 days. Figure 3 shows the degradation rate of the EGCG/Gels prepared under the different synthesis conditions (Table 1). The continuous shaking immediately disintegrated the products that did not contain EGCG and DMT-MM during synthesis, while simultaneous addition of DMT-MM, EGCG, and NMM at an adequate rate markedly altered the degradation speed of the EGCG/Gel. Approximately 70% of the EGCG/Gel retained its intact weight for 28 days. These results indicate that the present preparation system using DMT-MM, NMM and EGCG in water led to cross-linking of the Gel and EGCG via amide and ester linkages. When the Gel, which had many kinds of side chains, e.g., carboxy, amino, and hydroxy groups, was treated with DMT-MM under alkaline conditions, the triazinyl intermediates were first produced at the carboxy groups on the side chains. Then, the hydroxy group of the EGCG and Gel or the amino group of the Gel attached to the intermediate, forming the cross-links and supporting the EGCG.

### 2.3. Osteogenic Capability of EGCG/Gels

**Figure 4:**
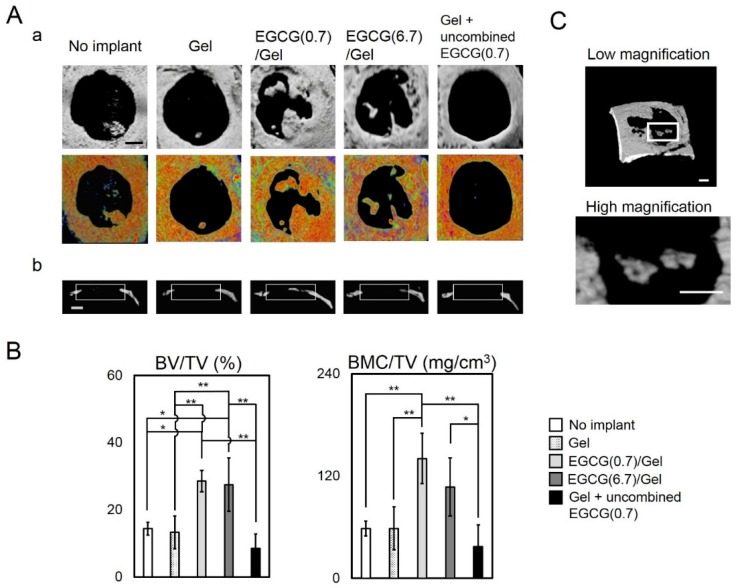
Micro computed tomography (μCT) and bone mineral density (BMD) images of Gel and EGCG/Gels and those quantitative data four weeks after implantation in the critical size defects of mouse calvaria. (**Aa**) Axial view of the μCT and BMD images of treated calvaria; (**Ab**) Lateral view of μCT images of treated calvaria. The width of the rectangles indicates the prepared bone defects; (**B**) Bone volume (BV) per total volume (TV) and bone mineral content (BMC) per TV in the defect. * *p* < 0.05; ** *p* < 0.01 (analysis of variance (ANOVA) with a Tukey-Kramer test). Data represent the mean with standard deviation (*n* = 3 per group); (**C**) High and low magnification of radiopaque parts isolated from the edge of the defect. Bars: (**A**,**C**) 1 mm.

**Figure 5:**
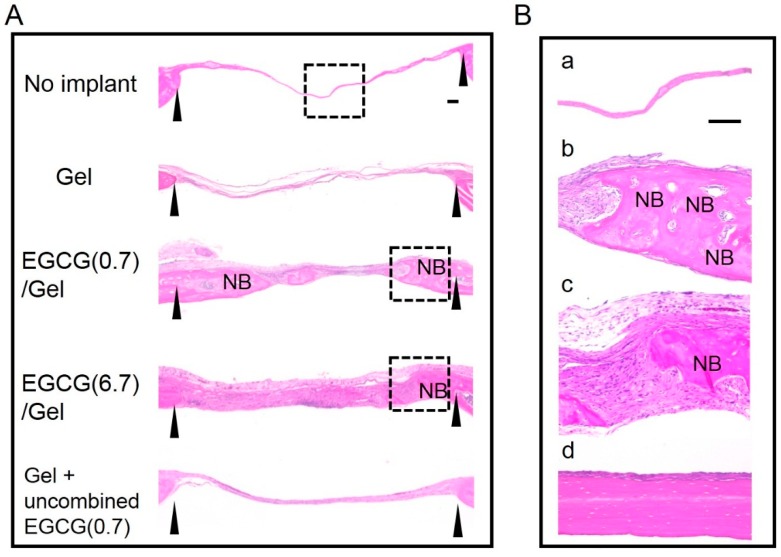
Images of the sections at four weeks after hematoxylin and eosin staining. (**A**) Overview image. Interval of arrowheads: the critical size defect in a mouse calvaria. NB: newly formed bone. Dashed line square: magnified area; and (**B**) Magnified images. (**a**) No implant; (**b**) EGCG (0.7)/Gel; (**c**) EGCG(6.7)/Gel and (**d**) intact bone of the calvaria without any treatment. Bars: 100 μm.

To verify whether the EGCG/Gel shows osteogenic capabilities *in vivo*, we implanted Gel and EGCG/Gels in critical size defects of mouse calvaria [32]. Of the EGCG/Gels, we selected EGCG(6.7)/Gel and EGCG(0.7)/Gel, which presented sponge shape similar to that of Gel, but contained different amounts of EGCG, to integrate the implant condition because high EGCG content yielded Gels with powder-like structures (Sample number 8 at Table 1). It is well known that the morphology of implant materials influences its osteogenic capability [33]. Figure 4 shows micro computed tomography (μCT) and bone mineral density (BMD) images of the defects treated with samples and the resulting quantitative data. After the four-week implantation, there were subtle differences in the defects treated with the Gel only and the no implant, while the radiopacity of the defects markedly increased in those with EGCG/Gels. Additionally, to verify the effect of conjugation of EGCG to Gel, we implanted (Gel + uncombined EGCG(0.7)) in which EGCG was added to Gel sponge just before implantation but was not conjugated with Gel. The Gel + uncombined EGCG(0.7) presented less radiopacity than the Gels combined with EGCG (Figure 4A). Coincident with the above radiopacity, the bone volume per total volume (BV/TV) and bone mineral content per TV (BMC/TV) of the defects treated with the EGCG/Gels were significantly higher than those of the no implant, Gel, and Gel with uncombined EGCG (Figure 4B). When we cautiously evaluated the radiopacity of the defect treated with the EGCG (0.7)/Gel, some radiopacity parts in the defect seemed to be separated from the edge of the defect (Figure 4C). Histological evaluation using hematoxylin and eosin (H–E) staining was performed to confirm whether the above radiopacity was newly formed bone (Figure 5). Thin fibrous tissues occupied the defects treated with the Gel, no implant, and Gel with uncombined EGCG. Meanwhile, as with the intact calvaria (Figure 5Bd), bony structures containing osteocytes could be observed from the tissue reconstructed by the EGCG/Gel implantations (Figure 5Bb,c). The newly formed bone partially covered the EGCG/Gels at the edge of bone defect, suggesting that the complexes have the potential of osteoconductivity (Figure S1).

### 2.4. Effects of EGCG/Gels on Osteoblast Differentiation

**Figure 6:**
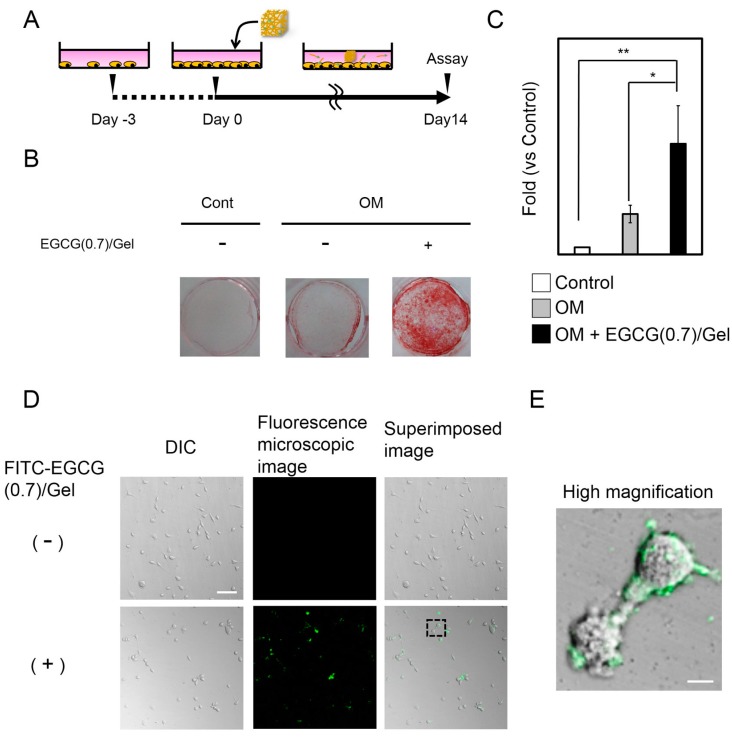
Effects of EGCG/Gels on the osteoblastogenesis of D1 cells *in vitro*. (**A**) Scheme of the experiment; (**B**,**C**) Alizarin red staining and its quantification data for the D1 cells treated with/without EGCG/Gels; (**A**–**C**) D1 cells were seeded at 1 × 10^5^ cells/well in a 24-well plate. On the prescribed date, the EGCG/Gel was added to the media. The mineralized matrix was stained with alizarin red S on Day 14. Cont: Control medium. OM: Osteogenic medium. + designated as 600 μg EGCG (0.7)/Gel. * *p* < 0.05, ** *p* < 0.01 (ANOVA with a Tukey-Kramer test). Data represent the mean with standard deviation (*n* = 3 per group); and (**D**,**E**) DIC and fluorescence microscopy images of the cells treated with FITC-labeled EGCG/Gel at low and high magnification. D1 cells were seeded at 1000 cells/well in 96-well plates. After overnight incubation, the cells were treated with approximately 20 μg of FITC-labeled EGCG (0.7)/Gel for 24 h. Dashed line square: magnified area. FITC-labeled EGCG/Gel adsorbed onto the cell surface of the D1 cells. Bars: (**D**) 100 μm and (**E**) 10 μm.

We further evaluated the mechanisms of the osteogenic capability on the EGCG/Gel using EGCG (0.7)/Gel and a mouse mesenchymal stem cell line (D1 cell) *in vitro* (Figure 6). The EGCG (0.7)/Gel were selected due to their high osteogenic capability in *in vivo* experiments (Figure 4 and Figure 5). The intensity of the alizarin red staining representing bone nodules, a well-known marker of later osteoblastogenesis, was stronger for the cells treated with the EGCG (0.7)/Gel compared with those treated with the control or osteogenic medium (OM) only (Figure 6A–C), suggesting that osteoblastogenesis of D1 cells was induced by EGCG/Gel administration (Figure 6). A previous study reported that D1 cells effectively differentiated into osteoblasts when treated with EGCG *in vitro* [25]. The results indicate the possibility that some mesenchymal stem cells existing in the bone defect may partially be involved in the bone formation induced by the EGCG/Gel.

### 2.5. Adsorption of EGCG/Gel to the D1 Cells

We next investigated the mechanism by which the EGCG/Gel activated the osteoblastogenesis of D1 cells using the FITC-labeled EGCG/Gel. After a 24 h treatment with the FITC-labeled EGCG/Gel, the fluorescent material adsorbed onto the D1 cell surface rather than into the cytoplasm (Figure 6D,E). No obvious changes could be observed within 72 h (Figure S2).

## 3. Discussion

Despite the broad consensus on the use of catechin as a health-promoting agent, the effective methods to deliver EGCG for local bone formation *in vivo* are still under consideration. The present study demonstrated that the conjugation of EGCG to the Gel delayed degradation of the complex, leading to enhancements in bone formation compared with the Gel alone or the Gel with uncombined EGCG. *In vitro* analysis revealed that the EGCG/Gel possessed the capability to induce osteoblastic differentiation of the mesenchymal stem cell line (D1 cells); this effect might be partially due to the accumulation of the EGCG/Gel on the cell membrane.

In the present study, we applied an aqueous chemical synthesis rather than conventional synthetic methods using organic solvents in the view of green sustainable chemistry [34,35]. It is important to replace toxic organic solvents with low-environmental-burden solvents, such as water, supercritical fluid, and ionic liquids, in order to prevent pollution of the environment and to avoid accidents. EGCG/Gels, which formed cross-links and supported EGCG in the Gel, were readily synthesized in water by using DMT-MM. The present simple and environment-friendly synthetic method is applicable not only to catechin analogs, such as flavonols, but also to other bioactive substances with hydroxy groups.

DMT-MM should have facilitated the chemical bonding between the amino acids in the Gel [36]. Nevertheless, this cross-link shows negligible effects on attenuating degradability (Figure 3). Meanwhile, the conjugation of the EGCG to the Gel remarkably delayed the degradation of the EGCG/Gel for up to 28 days, indicating that EGCG effectively worked as a cross-linker of the EGCG/Gel. There is a consensus that adequate stiffness is a key property for scaffolds to enhance cell adhesion, spreading, proliferation, and migration, eventually leading to bone formation [33]. Various chemical, enzymatic, and physical cross-linking methods have been applied to stabilize Gels, including genipin, aldehydes, UV light, dehydrothermal methods, *etc.* [37]. As with the other chemical cross-linking agents, the addition of the EGCG should stabilize the mechanical strength of the EGCG/Gel, which would partially contribute to the enhancement of bone formation in the present study.

To explore further osteogenic mechanisms of the EGCG/Gel independent of the anchorage dependence of a scaffold, we added the EGCG/Gel after cell seeding *in vitro* (Figure 6A). Disintegrated EGCG/Gels potently induced osteoblastogenesis in D1 cells without the anchorage dependence (Figure 6), as is coincident with the previous study using EGCG alone with D1 cells [25]. The results suggest that the EGCG/Gel works not only as a scaffold, but also as a stimulant for the cells in the bone defect after degradation. So far, we have not been able to verify which cells in the bone defect are preferentially stimulated by the EGCG/Gel to induce bone formation. However, a previous study reported that EGCG exhibits a negative effect on the osteoblastic differentiation in a murine osteoblastic cell line [26]. Our results may indicate that the EGCG/Gel preferentially stimulated some immature stem cells, leading to induce bone formation *in vivo*.

EGCG (0.7)/Gel presented potent osteogenic capabilities compared with the Gel alone *in vivo* (Figure 4 and Figure 5). Temporal supplementation of EGCG to the Gel (Gel + uncombined EGCG(0.7)) apparently hindered bone formation, suggesting that a long-term treatment with EGCG might be an effective strategy to induce robust bone formation. In addition to these results, Chen *et al.* reported that long-term treatment with EGCG has enhanced gene expression of osteogenic genes, alkaline phosphatase activity, and mineralization [25]. Our results show that the EGCG/Gel disintegrated gradually (Figure 3), and a fraction of the EGCG/Gel strongly absorbed onto the cellular membrane (Figure 6). It is well known that the amino acid sequence Arg-Gly-Asp (RGD) in a Gel has the potential to attach to cells via the RGD-integrin interaction [29,38]. The Gel in the EGCG/Gel might work as an anchor for the conjugated EGCG, enhancing the osteogenic capability of the EGCG/Gel, even *in vivo*.

To date, 100–200 μg of EGCG has had a significant effect on inducing bone formation [19]. In contrast, the EGCG (0.7)/Gel, containing only 4.2 μg of EGCG, seemed to be sufficient for inducing osteogenic capability in the present study. The above-mentioned anchoring reaction might contribute to the reduction in the essential dose of EGCG. However, further cautious examination, especially in view of the long implantation and signal pathways, would be imperative. Additionally, we could not detect the exact amount of EGCG released from the EGCG/Gels both in cell culture system and in the animal experiment because of interference from impurities containing phenol. The basic data will be valuable for exploring new materials. Further evaluation of the material would be imperative to prevent misuse of these materials in bone regenerative therapy.

## 4. Experimental Section

### 4.1. Chemicals

Gel from porcine skin was purchased from Sigma-Aldrich Co. LLC. (St. Louis, MO, USA). EGCG was purchased from BioVerde Inc. (Kyoto, Japan). DMT-MM and NMM were purchased from Tokyo Chemical Industry Co., Ltd. (Tokyo, Japan) and Nacalai Tesque Inc. (Kyoto, Japan), respectively. FITC-NCS was purchased from Dojindo Laboratories Co., Ltd. (Kumamoto, Japan).

### 4.2. Aqueous Chemical Synthesis of EGCG/Gels

Gel (100 mg) was dissolved in warm water (5 mL) at 50 °C. After the solution was cooled to room temperature, the solution with or without NMM, EGCG, and/or DMT-MM was stirred for 24 h at room temperature in the dark. The products were purified by dialysis (Spectra/Por7 MWCO 1000, Spectrum Labs, Rancho Dominguez, CA, USA) in water in the dark. The resulting solution was lyophilized by DC800 (Yamato Co., Ltd., Tokyo, Japan) in ϕ5 mm silicon tubes to produce the EGCG/Gel (Figure 1 and Figure 2). After lyophilization, the EGCG/Gels and Gels were dissected at approximately 2 mm height and stored in 4 °C in the dark until use.

### 4.3. Synthesis of FITC-Labeled EGCG/Gel

A mixture of 2 mg of EGCG(0.7)/Gel, 1 *w*_t_ % FITC-NCS DMSO solution (20 μL), and water (120 μL) was stirred for 24 h at room temperature in the dark. The product was purified by dialysis (Spectra/Por 7 MWCO 1000) in water in the dark. The resulting solution was used for the *in vitro* experiments.

### 4.4. Characterization of Gel and EGCG/Gels

The presence of EGCG in EGCG/Gels was evaluated using the Attenuated total reflection Fourier transform infrared spectroscopy (Perkin Elmer Spectrum GX, PerkinElmer, Waltham, MA, USA). The samples were analyzed over a range of 4000–750 cm^−1^ with 4 cm^−1^ resolution. The morphology of the Gel and EGCG/Gels was observed by FE-SEM (S-4000, Hitachi, Tokyo, Japan).

### 4.5. Robustness of EGCG/Gels

To evaluate the robustness of EGCG/Gels, the complexes (50 mg) were shaken (70 times/min) in water (15 mL) at room temperature. At the end of the shaking, the remaining EGCG/Gel was washed with water and lyophilized. The residual ratio was calculated as follows: (the weight of the remaining samples/the weight of the intact samples) × 100.

### 4.6. Animal Experiments

All animal experiments strictly followed the guidelines approved by the Local Ethics Committee of Osaka Dental University (Approval No.13-12002, 4 February 2014). Two disks of samples were implanted into critical-sized defects (ϕ4.2 mm) in 9–10-week-old ICR mice for up to 4 weeks. The critical size defects were prepared as reported previously [39]. No implantation was used as a negative control. (Gel + uncombined EGCG(0.7)) was prepared as follows: (1) Gels, hydrothermally treated at 150 °C for 24 h, were prepared to obtained crosslinked-Gel sponge; (2) Equal amounts of EGCG to that of EGCG (0.7)/Gel were dissolved in water and instilled into the hydrothermal-treated Gel before implantation. To analyze the microstructure of the newly formed bone, the calvaria, with implanted samples, were examined by μCT scanning (SMX-130CT, Shimadzu, Kyoto, Japan). The bone specimens were scanned continuously in 35-μm increments and yielded 476 slices, with a 48-kV tube voltage and a 70-μA tube current. The voxel size was 35 μm × 35 μm × 35 μm. After scanning, the image data were transferred to a workstation and were reconstructed in a 3D image analysis system (TRI/3D-Bon, Ratoc System Engineering, Tokyo, Japan). The BV and TV in prepared defect was evaluated to calculate BV/TV (%). In addition to bone volume, we also quantified the BMC at the level of calcified bone tissue using cylindrical phantoms containing a hydroxyapatite (HA content: 200 to 800 mg/cm^3^). As for the histological evaluation, three mice were used in each group. The treated mice were dissected at 4 weeks and the calvaria were fixed with 4% paraformaldehyde in 0.1 M phosphate buffer. The samples were decalcified, dehydrated, and embedded in paraffin. Thin sections (5 μm in thickness) were prepared and stained with H–E.

### 4.7. Cell Maintenance

The mouse mesenchymal stem cell line (D1 ORL UVA [D1], D1 cell, CRL-12424) derived from bone marrow, were purchased from American Type Culture Collection (ATCC, Manassas, VA, USA). D1 cells represent specific mesenchymal stem cell surface markers and are capable of differentiating into osteogenic and adipogenic stem cells [40]. The cell lines were maintained at subconfluence in Dulbecco’s Modified Essential Medium (DMEM), supplemented with 10% fetal bovine serum (FBS) and 1% antibiotics in a 5% CO_2_ incubator at 37 °C. The cells were used for assays at passages 3 to 6.

### 4.8. Effects of EGCG/Gel for Osteoblastogenesis

To evaluate the osteoblastogenesis of D1 cells, the cells were cultured in the following media with/without EGCG (0.7)/Gels: control medium (Cont: used as a negative control), basal DMEM with FBS and antibiotics; OM, control medium containing osteogenic factors (50 μM ascorbic acid 2-phosphate and 10 mM β*-*glycerophosphate). The calcified deposition was evaluated using alizarin red staining and its quantitative analysis. Briefly, cell cultures were washed with phosphate-buffered saline (PBS) and fixed with 95% ethanol. After incubation for 10 min, the cultures were stained with alizarin red S (Sigma-Aldrich Co. LLC) for 30 min, and excess dye was removed using distilled water. Images were captured using Canon A495 camera (Canon, Tokyo, Japan). Quantitative data of alizarin red staining were obtained as follows: (1) Ten percent formic acid was added to the stained samples and gently shook for 10 min to dissolve the deposit with dye; (2) The optical density of the supernatant was measured using a microplate reader (SpectraMax M5, Molecular Devices, Sunnyvale, CA, USA) at 415 nm. Relative alizarin red staining level = Absorbance of sample/absorbance of benchmark. Cells treated with control were used as the benchmark.

### 4.9. Adsorption of FITC-Labeled-EGCG (0.7)/Gel onto a Mesenchymal Stem Cell Line

D1 cells were seeded at 1000 cells/well in a 96-well plate. After overnight incubation, the cells were treated with 20 μg of FITC-labeled EGCG (0.7)/Gel for up to 72 h. The cells were gently washed with PBS two times before images were captured. All immunofluorescence images were captured using a confocal laser scanning microscope (Zeiss LSM700, Carl Zeiss Microscopy, Jena, Germany).

### 4.10. Statistical Analysis

Statistical significance was evaluated using a one-way analysis of variance, followed by a Tukey-Kramer test. Microsoft Excel software statistic package was used for the calculations.

## 5. Conclusions

In the present study, we demonstrated that EGCG-conjugated Gels presented potent osteogenic capability compared with that of Gels without EGCG in critical size defects of mouse calvaria. Gel with uncombined-EGCG hardly induced bone formation even when it contained EGCG equal to that in the EGCG (0.7)/Gel. Additionally, the EGCG/Gel significantly induced osteoblastogenesis for mouse mesenchymal stem cell line D1 cells within 14 days, suggesting that the EGCG/Gel might stimulate the mesenchymal stem cell in the bone defect. The above activation seems to be partially due to the sustained adsorption of the EGCG/Gel onto the surface of the D1 cells. These results indicate that the controlled release system of the polyphenol at a local site is adequate, and provides a usable strategy to induce bone formation.

## Supplementary Materials

Supplementary materials can be found at http://www.mdpi.com/1422-0067/16/06/14143/s1.
