# Supplementary material for: Local Controlled Release of Polyphenol Conjugated with Gelatin Facilitates Bone Formation
Source: Int J Mol Sci. 2015 Jun 23;16(6):14143–57. doi: 10.3390/ijms160614143 (PMC4490544; doi:10.3390/ijms160614143)
Supplement: Supplementary file 1 [file ijms-16-14143-s001.pdf]

Supplementary Information

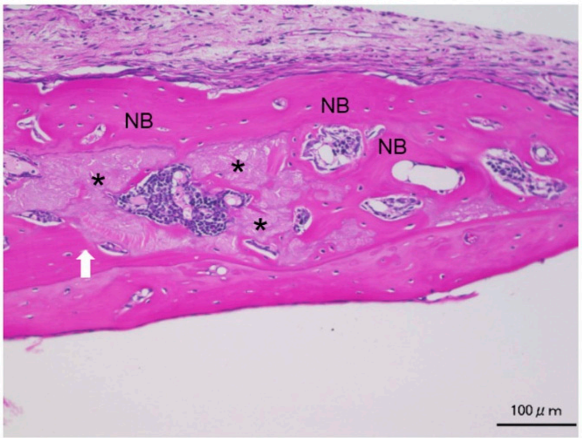

Figure S1. Image of EGCG(0.7)/Gel section at 4 weeks after hematoxylin and eosin staining. NB: newly formed bone. Asterisks: residual complex. White arrow: the edge of created bone defect.

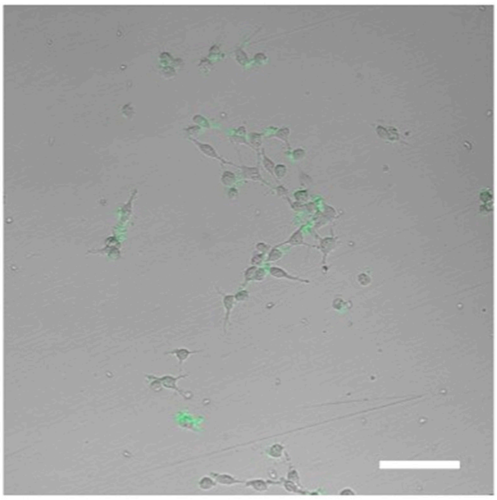

Figure S2. Superimposed image of the cells treated with FITC-labeled EGCG (0.7)/Gel for 72 h. D1 cells were seeded at 1000/well in 96 well plates. After overnight incubation, the cells were treated with approximately 20 μg of FITC-labeled EGCG (0.7)/Gel. The FITC-labeled EGCG (0.7)/Gel adsorbed onto the cell surface of the D1 cells. Bar = 100 μm.
